# Supplementary material for: Lipid Biomarkers as Predictors of Diastolic Dysfunction in Diabetes with Poor Glycemic Control
Source: Int J Mol Sci. 2020 Jul 18;21(14):5079. doi: 10.3390/ijms21145079 (PMC7404098; doi:10.3390/ijms21145079)
Supplement: Supplementary file 1 [file ijms-21-05079-s001.pdf]

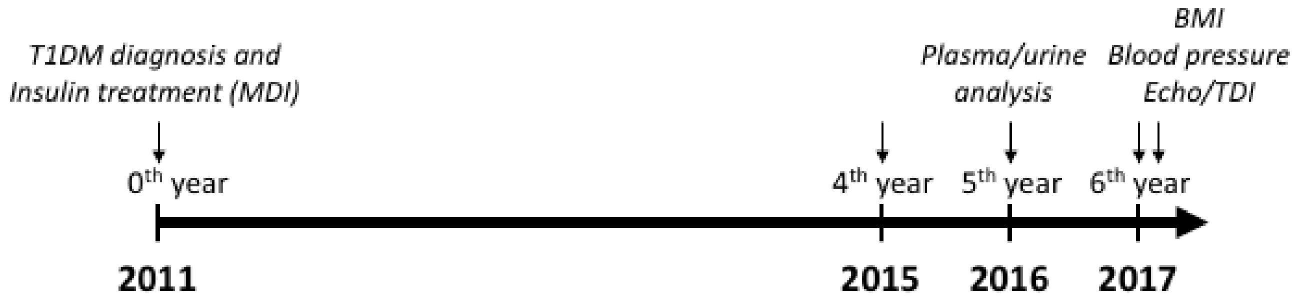

Figure 1. Schematic representation of the study. Seventy-eight young T1DM patients were diagnosed of T1DM in 2011 and treated with multiple daily injection (MDI) regimen of insulin for 6 years. In 2017, their plasma and urine parameters were retrospectively analysed at three stages (2015, 2016 and 2017), and then, blood pressure and cardiac function was evaluated by Echo-2D-Doppler and TDI.

Table S1. Blood pressure and systolic dysfunction did not associate with diastolic failure in uncontrolled T1DM. Systolic and diastolic blood pressures (SBP and DBP), and 2D-Echo/Doppler markers of systolic function did not correlate with the development of diastolic injury, either in girls and boys. FS, fractional shortening; EF, ejection fraction; LVEDVI, LV end-diastolic volume index.

	**T1DM Males (n= 30)**			**T1DM Females (n= 48)**		
	Non-ventricular dysfunction (*n* = 10)	Ventricular dysfunction (*n* = 20)	P value	Non-ventricular dysfunction (*n* = 24)	Ventricular dysfunction (*n* = 24)	*p* value
**SBP (mm Hg)**	116.8 ± 7.59	120.35 ± 8.99	0.29	110.66 ± 8.31	113.66 ± 10.17	0.26
**DBP (mm Hg)**	73.8 ± 8.37	77.2 ± 7.23	0.25	73.66 ± 8.44	78.5 ± 8.78	0.06
**2D-Echo/Doppler**						
FS (%)	41.5 (8.75)	43.5 (5.75)	0.61	39.0 (11.5)	39.5 (8.0)	0.27
EF (%)	75.0 (16.25)	78.5 (10.75)	0.53	72.0 (17.0)	74.5 (15.0)	0.22
LVEDVI (mL/m^2)	73.0 (6.0)	71.0 (4.75)	0.30	73.0 (3.75)	71.0 (5.0)	0.06
